# Monthly Summary

**Published:** 1882-04

**Authors:** 


### MONTHLY SUMMARY.

---

*Where Nitrogen is Found.*—Everywhere, then, where air is, there is nitrogen. Not only in the atmosphere as it surrounds the whole earth, but in the waters of springs, streams, and the ocean which dissolves air and holds it, and in the soil which the air likewise permeates, does the ærial nitrogen occur. But there is a great deal of nitrogen besides that in the air. Nitrogen forms a part of every plant, from the grass to the oak, and is in every organ, in root, stem, leaf, flower, fruit, and seed. It occurs in the body of every animal, and in every part of the body. Neither plants nor animals could live or even exist without it. It is an essential element of all our foods, and the costliest component of fertilizers. Its compounds are in earth, and air, and sea; in very fertile soil; indeed no soil could be fertile without them.

I have just spoken of compounds of nitrogen, and ought to explain the difference between nitrogen in the free or uncombined state, and in compounds. The nitrogen that makes up the bulk of the air, is in what chemists call the free state. The minute particles, atoms, as they are called, are not united to those of any other element, they exist by themselves. But they are capable of combining with other elements to form compounds. Thus an atom of nitrogen unites with three atoms of hydrogen to form a compound called ammonia, and two atoms of nitrogen combine with five atoms of oxygen to form a compound called nitric acid. So long as the nitrogen remains free, neither plants nor animals can make the most complete use of it, but in its compounds it can be directly used as food by both animals and plants. And the compounds of nitrogen are employed in a great variety of ways to supply the wants of man.—*Prof. Atwater, in American Agriculturist.*

---

*A New Method of Embalming Bodies and Preserving Tissues.*—Dr. Virodtzeff (*Balsamirovvanie,* pp. xi., 164, St. Petersburg, 1881) recommends the following preparation as an efficient agent in the embalming of bodies and the preservation of tissues:

| | | |
|---|---|---|
| Thymol............................... | 5 | parts. |
| Alcohol............................... | 45 | " |
| Glycerine............................ | 2,160 | " |
| Water................................ | 1,080 | " |

It is cheap, innocuous, free from unpleasant odor, possesses the property of keeping the body soft, elastic, fresh and life-like, and does not ruin instruments. Thymol is selected as being superior to other antiseptics, and glycerine is added, both on account of its own preservative qualities and to retard the evaporation of the fluid. For the preparation of tissues the same solution is employed. If the cadaver be quite lean, or the tissues very delicate, equal parts of water and glycerine (1,620 each) are combined with the above quantities of thymol and alcohol. To inject a body, half its weight of the fluid is necessary. A properly embalmed cadaver may be preserved indefinitely under ordinary circumstances, gradually shrinking and mumifying without putrefaction. Specimens are either to be injected with or macerated in this fluid. Maceration must not be too prolonged—the appearance of the specimen should act as a guide. The part, after having been thoroughly cleansed in water, and prepared, may then be exposed for months to the air without losing its consistency, form, and color. Permanent specimens may be enclosed in a hermetically sealed glass vessel containing a little of the same solution. Dr. Peabody has used this preserving fluid, with excellent results, in the New York Hospital Museum.

---

*The Teeth in Diabetes.*—Dr. Magitot has after an examination of many diabetics, come to the following conclusions: First: Examination of the mouths of diabetics, furnishes a constant symptom of the disease. Second: This symptom is a lesion of the alveolar border which may be designated as an alveolar osteo-periostitis. Third: This manifestation appears at the onset of the disease, persists during its course and can, in consequence, be considered a pathognomonic symptom. Fourth: This alveolar affection, considered as a symptom of diabetes, presents three periods. Its first period is that of simple deviation of the teeth. Its second period is that of loosening of the teeth and alveolar catarrh. Each of these periods is in relation to the phase of the constitutional disease. The third period, that of the falling out of the teeth, corresponds to a more advanced state of glycosuria. Beside this last symptom there may occur, if the patient lives long enough, an osseous resorbtion which may, or may not, be consecutive to a gangrene of the gums. The appearance of this latter complication is evidence of a critical stage of the disease, as it ordinarily ushers in its fatal termination. The value, as a symptom, of the first stage of dental changes, remains to be determined. It must be obvious, however, that it can only occur in the more chronic forms of glycosuric diabetes.—*Exchange.*

